# Age and sex differences in numerical responses, dietary shifts, and total responses of a generalist predator to population dynamics of main prey

**DOI:** 10.1007/s00442-020-04607-x

**Published:** 2020-02-01

**Authors:** Giulia Masoero, Toni Laaksonen, Chiara Morosinotto, Erkki Korpimäki

**Affiliations:** 1 Section of Ecology, Department of Biology, University of Turku, 20014 Turku, Finland; 2 Natural Resources Institute Finland (Luke), Turku, Finland; 3 Novia University of Applied Sciences, Bioeconomy Research Team, Raseborgsvägen 9, 10600 Ekenäs, Finland

**Keywords:** Boreal forest, Food hoarding, Functional response, Intraspecific variation, Predator–prey interactions, Starvation risk, Vole cycle

## Abstract

Fluctuations in the abundance of main prey species might shape animal communities, by inducing numerical responses and dietary shifts in predators. Whether numerical responses and dietary shifts differ among individuals of different age and sex has so far gained little attention. These differences could affect how much predators consume main and alternative prey, thus causing variation in predation pressure on main and alternative prey species. We studied the effect of fluctuating main prey abundance (voles) in autumn on the age and sex composition of a food-hoarding population of Eurasian pygmy owls *Glaucidium passerinum* (327 individuals), and on the species composition of their food stores in western Finland during 2003–2017 (629 food stores). Numbers of yearlings (< 1-year old) of both sexes and adult (+ 1-year old) females increased with increasing vole abundance. During low vole abundance, adult owls stored more small birds and less small mammals than yearlings. Females stored more small mammals than males and showed a tendency to store less birds. The amount of consumed birds (the most important alternative prey), and in particular of crested, willow, great, and blue tits, increased with low vole densities. Our results show that numerical, functional, and total responses of pygmy owls, and probably also other vertebrate predators, to the availability of the main prey in winter are shaped by the age and sex composition of the predator population, which both show large spatio-temporal variation in boreal forests.

**Electronic supplementary material:**

The online version of this article (10.1007/s00442-020-04607-x) contains supplementary material, which is available to authorized users.

## Introduction

Predator–prey interactions are considered to be one of the major forces determining the structure and dynamics of animal communities (Vermeij 1994; Abrams 2000; Salo et al. 2010). Natural systems are rarely composed by only two interacting species, and the presence of a predator can influence and shape the whole prey community (Winnie and Creel 2017). Important features that characterize predator–prey interactions are how predators respond both numerically and functionally to population dynamics of prey (Solomon 1949). The numerical response describes how predator abundance varies in relation to prey abundance and is induced by changes in natality, mortality, emigration, and immigration within the predator population (e.g., Solomon 1949; Korpimäki and Norrdahl 1989, 1991; O’Donoghue et al. 1997; Salamolard et al. 2000). The functional response of predators describes how the rate of prey capture varies in relation to prey abundance (e.g., Oaten and Murdoch 1975). Predators can show dietary shifts and switch to alternative prey species when the abundance of the main prey decreases (e.g., Korpimäki 1987a; Korpimäki and Norrdahl 1989, 1991; O’Donoghue et al. 1998). With some exceptions (Korpimäki et al. 1991; O’Donoghue et al. 1997, 1998), the vast majority of studies on dietary shifts and numerical responses of vertebrate predators have been conducted during the reproductive season. Investigating predator populations and their responses to main prey abundance during autumn and winter in boreal regions could provide useful insight on predator–prey and community dynamics, due to the restricted food resources and harsh climatic conditions of the boreal winter.

How traits of predators, such as age and sex, can influence numerical responses and dietary shifts to changes in prey abundance is a neglected question. While there are many studies analysing inter-specific differences in functional responses of vertebrate predators to varying densities of prey populations (e.g., Korpimäki and Norrdahl 1991; Korpimäki et al. 1991; O’Donoghue et al. 1998; Therrien et al. 2014), very little is known about their intraspecific age and sex differences. Individuals of different sex and age classes can have different diets due to differences in behaviour, size, and hunting skills, or because of foraging segregation. Sex differences in the hunting skills and behaviour have been shown, for example, in many avian predators (Earhart and Johnson 1970; Keynan and Yosef 2010; Korpimäki and Hakkarainen 2012). In birds of prey, different behaviour and nutritional needs, together with differences in body size (i.e., reversed sexual size dimorphism), can lead to different diets in males and females (Newton and Marquiss 1982; Hakkarainen and Korpimäki 1991; Lee and Severinghaus 2004). An age effect on breeding and hunting skills can be due to young individuals being either constrained by their inferior skills or refraining from using maximal effort, or due to their cohort being composed of lower quality individuals no longer present in the older age groups (Curio 1983; Forslund and Pärt 1995). Foraging segregation might, therefore, rise from the different hunting skills and experience between juveniles and adults (Marchetti and Price 1989; Wunderle 1991).

Previous studies on functional responses have examined either differences in relation to predator sex (Parajulee et al. 1994) or social status (Nilsen et al. 2009). Whereas differences in numerical response have been looked at in relation to age and/or sex in breeding (e.g., Rohner 1996) and in wintering birds of prey (e.g., Village 1985; Côté et al. 2007; Korpimäki and Hakkarainen 2012). However, to our knowledge, previous studies have not investigated age and sex differences simultaneously, in both numerical and functional responses, and the resulting total response. Differences in the age and sex composition of the predator population and in their dietary shifts are important, also because it may reflect on how the predator impacts the prey community.

In Eurasian boreal forests, the multi-annual fluctuations in small mammal abundance (Krebs and Myers 1978; Hansson and Henttonen 1988) strongly govern the density and breeding success of mammalian and avian predators (Korpimäki and Norrdahl 1989; Korpimäki 1992; Lehikoinen et al. 2011b). Consequently, it can indirectly affect alternative prey species. The Eurasian pygmy owl (*Glaucidium passerinum*; hereafter pygmy owl) is the smallest avian predator in Europe and the only one that hoards large quantities of prey for the winter (Mikkola 1983; Solheim 1984a; Terraube et al. 2017). An increase in the main prey (voles) population induces higher numbers of breeding pairs (Solheim 1984b), and of prey items per food store in winter (Solheim 1984a; Suhonen et al. 1993; Terraube et al. 2017). Recently, we found that pygmy owls showed age- and sex-related variations in the total amount of food stored in relation to fluctuating vole abundance (Masoero et al. 2018). However, we did not find any study investigating sex or age differences in numerical responses and dietary shifts in relation to vole abundance. Understanding the dissimilarities in the total response arising from the different numerical responses and dietary shifts of the age and sex classes of the same predator species could be an important step to comprehend their potential impacts on prey community.

We first examined the variation in age and sex composition of a pygmy owl population in winter in relation to the natural abundance of their main prey (i.e., numerical response). Second, we investigated the variation in the number of various prey species in the food stores of yearlings and adults, as well as female and male pygmy owls, in relation to natural abundance of their main prey (i.e., dietary shifts). We made the following predictions:

1. Adult males of pygmy owls have usually not been observed during irruptive migratory movements (Lehikoinen et al. 2011a), and thus, their numerical response to fluctuating vole densities should be less pronounced than that of other individuals. On the other hand, we can expect a low number of first-year owls of both sexes and of adult females in the wintering population when the vole abundance is low, followed by an increment when vole abundance increases, due to more abundant food supply reducing intraspecific competition.
2. Due to differential mortality, first-year cohorts may be partially composed of lower quality individuals no longer present in the older age groups, and first-year owls may be less proficient hunters than adults due to lack of experience or hunting skills. Therefore, first-year owls can be expected to be less able to shift from main prey (voles) to alternative prey (small birds), and to store species that are easier to catch (i.e., small mammals instead of birds). Females can be expected to store bigger and heavier species (i.e., voles of the genus *Microtus*) more frequently than males because of their larger size, whereas males, supposedly more agile, may be better able to hunt, and thus hoard, more birds. The aforementioned differences between age classes and sexes in terms of alternative prey are predicted to be greater in years of low vole abundance, when pygmy owls should shift from hunting main prey to scarcer alternative prey (small birds, shrews, and mice).
3. Combining the predictions for the two responses, the autumn/winter population structure of pygmy owls in terms of age and sex classes is expected to vary according to main prey abundance, as is the consumption rate of main and alternative prey species. We expect more yearlings and females in the population in years of high vole abundance, resulting in larger consumption rates for the main prey (voles). In years of vole scarcity, however, fewer owls, mainly adult males, may be present in the population, resulting in a shift in prey consumption towards birds. Numerical responses and dietary shifts of individuals of different age and sex classes according to the abundance of the main prey would, therefore, determine among-year variations in the consumed prey (total response).

## Materials and methods

### Study area, predator, and prey species

The study area is situated in the vicinity of Kauhava (Southern Ostrobothnia, 63°N, 23°E), western Finland, and covers approximately 1000 km^2^ of managed forests, mainly composed of Scots pine (*Pinus sylvestris*), Norway spruce (*Picea abies*) and in smaller proportions some deciduous trees, interspersed with agricultural land (Morosinotto et al. 2017a). The proportion of the managed forests is approx. 70% and the proportion of agricultural fields is 25% (the rest is mainly peatland bogs and settlement areas). Agricultural fields are interspersed all over the study area. The data were collected from 2003 to 2017 in 305 sites (hereafter ‘forest sites’), each provided with two nest-boxes for pygmy owls (for further details on the study system, see Terraube et al. 2017, Masoero et al. 2018). To prevent other species of owls from entering the boxes, the diameter of the entrance hole of nest-boxes was 45 mm, which corresponds to a cavity excavated by the three-toed woodpecker *Picoides tridactylus* (Solheim 1984a).

The pygmy owl is a small diurnal avian predator that inhabits old and mature coniferous forests of Eurasia (Schönn 1980; Mikkola 1983; Strom and Sonerud 2001; Barbaro et al. 2016). The species stores large quantity of food in natural tree cavities or nest-boxes for a few weeks or months during late autumn and winter (Solheim 1984a; Terraube et al. 2017). Pygmy owl diet is related to prey availability and can, therefore, differ according to season and geographic area (Schönn 1980; Mikkola 1983; Ekman 1986; Schulenburg and Wiesner 1986). In North Europe, pygmy owls mostly prey upon bank voles (*Myodes glareolus*), voles of the genus *Microtus* (the field vole *M. agrestis*, and the sibling vole *M. rossiaemeridionalis*; hereafter ‘*Microtus* voles’), shrews (the common shrew *Sorex araneus*, the pygmy shrew *S. minutus*), mice (the Eurasian harvest mouse *Micromys minutus*, the house mouse *Mus musculus*), and small birds, usually passerines, with body mass < 60 g (Kellomäki 1977; Halonen et al. 2007). The two main prey groups of the pygmy owl are the bank voles and the *Microtus* voles. The first one is more commonly found in forested areas, and the second one in more open areas, such as agricultural fields and clear-cut areas. Abundance of vole species in the study area fluctuates in 3-year cycles with 100–200-fold amplitude (Korpimäki et al. 2005). Among the avian prey species during autumn and winter, there are usually resident forest birds, such as tit species and goldcrests *Regulus regulus*.

### Data collection

Between late-October and mid-December, all the box sites were inspected twice to collect data on the prey composition of the food stores of pygmy owls. We calculated the total number of prey items of the main five groups of species by summing up the fresh prey items counted in the two visits done in autumn. The five main groups were: bank voles, *Microtus* voles, shrews, mice, and small birds. To avoid double counting, prey items in food stores were marked with tail clipping (mammals) or toe clipping (birds). We decided to analyse the data by considering the content of the single store and not of the sum of the stores hoarded by an individual. This measure was considered better in analyses of store contents, since it can be measured exactly, while the total number of prey hoarded by an owl would be less precise due to unknown stores (i.e., in natural cavities or in a box without an identified hoarder). We excluded from the analyses the cases in which it was not possible to identify the content of the store (7.5% of the total prey items), and in which the hoarder of the store was not identified with certainty or it remained unsexed (see “Results”).

In the study area, pygmy owls were trapped during the hoarding season using mostly nest-box traps. Captured owls were ringed with an aluminium leg ring for individual identification, their wing and tail lengths were measured, and they were weighted, sexed, and aged. Pygmy owls are sexually dimorphic, with females larger than males, and sex was therefore determined on the basis of wing length, tail length, and body mass (as in Masoero et al. 2018). The age was estimated according to wing moult (Lagerström and Syrjänen 1990), and individuals were divided in two classes: individuals at their hatching year (hereafter ‘yearlings’) and older individuals (hereafter ‘adults’). Since 2011, the owls were also provided with a Passive Integrated Transponder (PIT) tag implanted subcutaneously, which helped collecting data on the identity of the storing owl (hereafter ‘hoarder’). Both direct captures and the data collected with the PIT-tag method contributed to the data on the number of hoarding owls and on the identity of the hoarder.

Natural abundance of the main prey of pygmy owls (bank voles and *Microtus* voles) was estimated by snap trapping twice a year (early May and mid-September) at two sites situated in the western and central part of the study area. All the four main habitat types (spruce forest, pine forest, cultivated field, and abandoned agricultural field) were sampled. Fifty baited Finnish metal mouse snap traps were set at 10-m intervals in vole runways on each sample plot and were checked daily for 3 consecutive days. Thus, the area of a sample plot was 0.5 ha and the pooled trapping effort was 600 trap nights in both western and central parts of the study area. The results of the three-night trapping periods were then pooled and standardized as the number of animals caught per 100 trap nights, creating one autumn abundance index for each group (see Korpimäki et al. 2005 for further details). From previous studies (Huitu et al. 2003; Korpimäki et al. 2005), it is known that that densities of *Microtus* and bank voles fluctuate in synchrony in the study area and that the regional synchrony of vole population cycles extends up to 80 km, therefore covering the whole study area. The standardized abundances of bank voles and *Microtus* voles estimated from snap trappings were also pooled together in an overall ‘vole index’.

### Statistical analyses

Analyses were carried out using Generalised Linear Models (GLMs) and Generalised Linear Mixed Models (GLMMs), fitted using maximum likelihood (Laplace approximation), package lme4 (Bates et al. 2015). Count data were analysed with a Poisson likelihood family, corrected in case of overdispersion as quasi-poisson (GLMs—age and sex population structure) or negative binomial (GLMMs—food store composition), while the proportional data were analysed with a binomial family, corrected as quasi-binomial (Zuur et al. 2009). The package glmmADMB was used in case of negative binomial models (Skaug et al. 2016). All models and statistical analyses were done using R v. 3.4.3 (R Core Team 2019).

For the analysis on age and sex population structure, we used the number of wintering individuals of the four classes of pygmy owls in relation to the vole abundance index of the current autumn. Year was included in the initial model to allow for differences among years, but was not significant and removed during subsequent model selection. Within the model, non-independence of errors due to temporal autocorrelation was accounted for by the addition of an autoregressive term of order 1 (corAR1).

We modelled how composition of food stores varied with sex and age of the owl as well as with vole abundance in the current autumn. Both the exact number and the relative proportion (constructed with the ‘cbind’ command in R) of prey items, divided into the five main groups, were used as response variables. The bank vole and the *Microtus* vole abundance indices were used for the respective groups, whereas the pooled vole abundance index was used in the models for the other three groups of prey (birds, shrews, mice). For this part of analyses, only the abundance index in the autumn of the current year was used, since the content of the food stores closely reflects prey availability in the environment when the food is hoarded (Masoero et al. 2018). The nest-box identity, nested in the forest site (i.e., where each box pair was set), was included as random effect to account for spatial and temporal pseudo-replication (two nest-boxes available for each forest site and most boxes were used multiple times during the study). We controlled for repeated measures of the same individual (in case of more than one store in different boxes in the same year, or of recaptures between multiple years) using individual identity (the code of the owl ring) as random effect. We started with a full model (all the explanatory variables and the two-way interactions), but interactions were removed if not significant using backward stepwise selection (significance evaluated with the function ‘Anova’ in the package *car*).

To understand the total response of pygmy owls, the total weight and the total number of prey items consumed were estimated according to formula of Korpimäki and Norrdahl (1989). For each of the five main groups of stored prey items, we calculated the number of prey items consumed as follows:

$${\text{NPA}}_{\text{group}} { = }\frac{{{\text{C}}_{\text{AM}} \, \times \,{\text{PPA}}_{\text{AM}} \,{\text{ + C}}_{\text{AF}} \, \times \,{\text{PPA}}_{\text{AF}} \,{ + }\,{\text{C}}_{\text{YM}} \, \times \,{\text{PPA}}_{\text{YM}} { + }\,{\text{C}}_{\text{YF}} \, \times \,{\text{PPA}}_{\text{YF}} }}{\text{MWPA}},$$

where ${\text{C}}_{{{\text{age}} - {\text{sex}}}}$ (C_AM_, C_AF_, C_YM_, C_YF_, respectively) corresponds to the consumption (g) of each age and sex class during the food-hoarding season, and was calculated as the number of individuals of that age and sex class × daily food requirement of that sex class × length of the food-hoarding season (60 days). Daily food requirement was estimated as 40 g per day for males and 45 g per day for females. The mean daily food consumption of captive pygmy owls is 30 g (Glutz von Blotzheim and Bauer 1980), but at low temperatures (− 10°C), the daily food consumption was twice as large as during above 0°C temperatures (Scherzinger 1970). ${\text{PPA}}_{{{\text{age}} - {\text{sex}}}}$ corresponds to the percentage of prey group biomass in the food stores of that age and sex class during the food-hoarding season. MWPA corresponds to the mean weight (g) of prey animals (see Online Resource 1). With GLMs with quasi-poisson family, the estimated number of prey items consumed was then modelled in relation to the autumn vole abundance (the bank vole and the *Microtus* vole abundance indices were used for the respective groups, whereas the pooled vole abundance index was used in the models for the other three groups of prey).

The same analyses on the total response were carried out for the bird species most commonly found in the food stores (see Online Resource 1) to estimate the number of prey birds consumed by the pygmy owls. For each of the five bird species, the estimated number was then modelled in relation to the vole abundance of the current autumn using GLMs with quasi-poisson family (Online Resource 2).

## Results

### Composition of the predator population

**Fig. 1:**
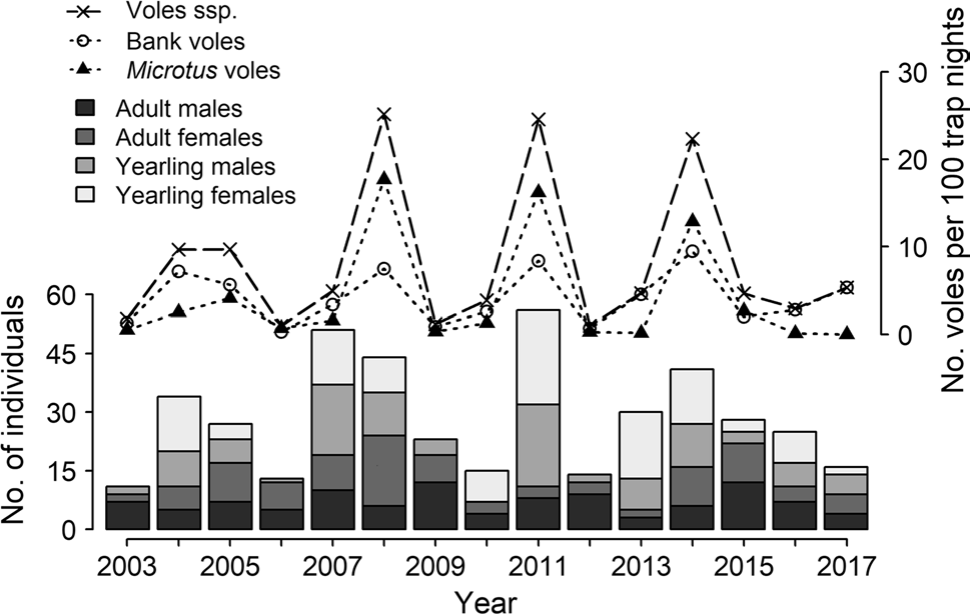
Among-year variation in the number of yearling, adult, male and female pygmy owls with a food store (barplot) and in the autumn vole abundance indices (No. of voles per 100 trap nights) for voles ssp. (dashed line), bank voles (dotted line and empty circle), and *Microtus* voles (dotted line and black triangle) in the study area from 2003 to 2017

A total of 344 pygmy owl individuals were identified, and ringed or recorded with pit-tag readers (150 males, 177 females, 17 unsexed) at 629 food stores during 2003–2017. The four age and sex groups showed wide inter-annual variation in numbers of individuals (Fig. 1). The number of adult males showed the lowest coefficient of variation (CV = 39.7%), while the numbers of adult females (65.5%), yearling males (85.4%), and yearling females (95.6%) showed wider between-year variations.

**Fig. 2:**
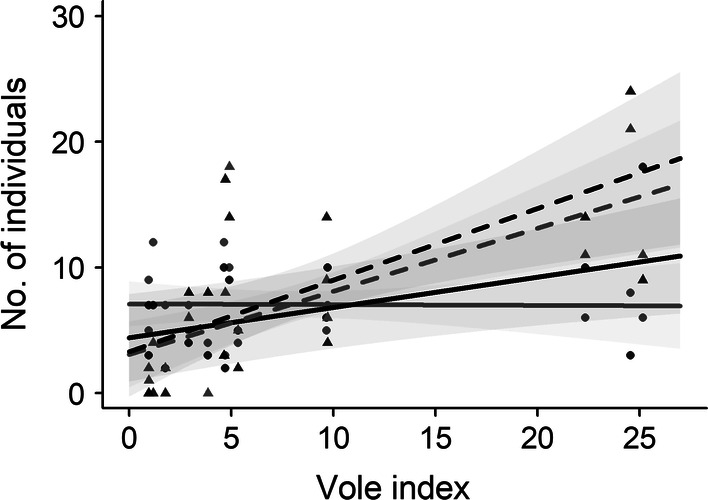
The regression line (with 95% CI; grey) of the number of food-storing individuals per year in relation to the vole abundance in the autumn of the current year for yearling (dashed line for the predicted values; triangles for the data) and adult (solid line, dots), female (black, CI in darker grey), and male (grey, CI in lighter grey) pygmy owls. All *P* values were significant (lower than 0.05), except for adult males (see Table 1)

The number of adult males did not show any obvious relationship with the vole abundance, whereas the numbers of yearlings of both sexes and of adult females were significantly positively correlated with the abundance of voles in the current autumn (Table 1, Fig. 2). The inclusion of a temporal correlation term allowed to estimate the correlation between residuals separated by 1 year (parameter ρ). The positive values assumed by parameter ρ for adult females indicated that the values of a particular year are positively related to preceding years. For yearlings of both sexes, however, it assumed negative values, reflecting the fact that the abundances go from high values in 1 year to low values in the next.

**Table 1:** GLMs analysing the annual variation in the number of pygmy owls with a food store in the four age and sex classes in relation to the vole abundance of the current year (vole index)

Response variable	Explanatory variable	Estimate ± SE	χ^2^	*P*	*ρ*
No. adult males	Intercept	7.100 **±** 0.912			− 0.3236
	Vole index	− 0.006 **±** 0.089	0.05	0.9451	
No. adult females	Intercept	4.404 **±** 1.777			0.4886
	Vole index	0.241 **±** 0.086	7.91	**0.0049**	
No. yearling males	Intercept	3.083 **±** 1.333			− 0.4339
	Vole index	0.502 **±** 0.133	14.31	**0.0002**	
No. yearling females	Intercept	3.296 **±** 1.822			− 0.3743
	Vole index	0.570 **±** 0.179	10.08	**0.0015**	

Note that the estimates are at log scale and significant *P* values are highlighted in bold (*P* < 0.05). *N* = 15 years, for a total of 327 individuals. The parameter ρ was estimated from the temporal correlation structure AR1 added to the model, and represents the correlation between the residuals of 1 year and the previous

### Variation in the composition of the food stores

A total of 17,838 prey items were found in 1084 food stores of pygmy owls during 2003–2017, of which a vast majority (93%) was mammals (Online Resource 1). The bank vole was the most frequent prey species in the stores (48% of prey number in food stores), followed by the two species of *Microtus* voles (29%), four shrew species (11%), 25 bird species (7%), and two mouse species (5%). The most abundant bird species in the food stores were the willow tit *Poecile montanus*, the great tit *Parus major*, the goldcrest, the Eurasian blue tit *Cyanistes caeruleus,* and the crested tit *Lophophanes cristatus* (Online Resource 1). In the 629 stores with an identified and sexed hoarder, 12,595 prey items were found, with the number of items per store ranging from one to 205 and a total store biomass ranging from three to 4129 grams.

**Fig. 3:**
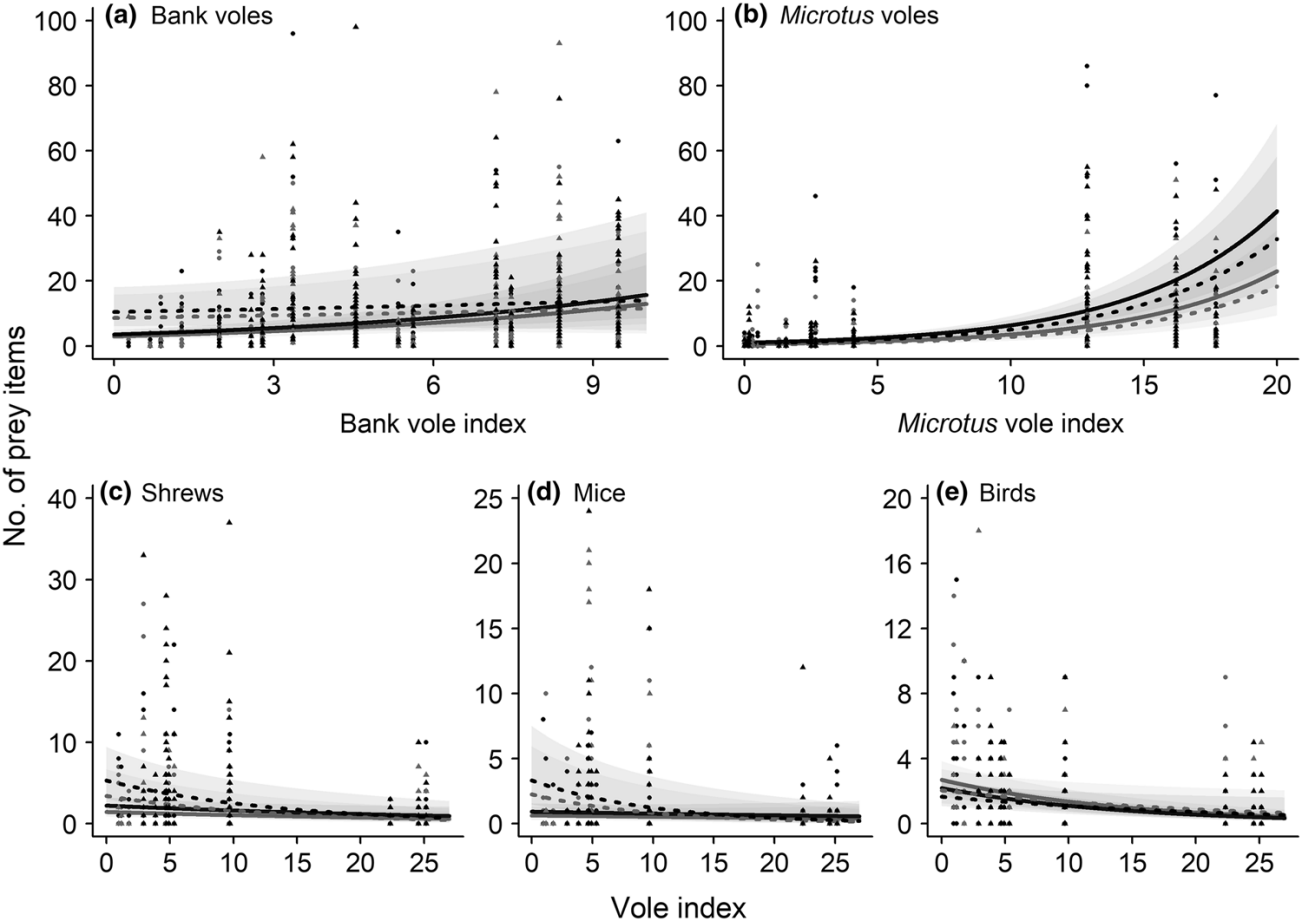
Predicted values (and 95% CI) of the number of prey items per single food store in relation to the vole abundance in the autumn of the current year for yearling (dashed line for the predicted values, triangles for the data) and adult (solid line, dots), female (black, CI in darker grey), and male (grey, CI in lighter grey) pygmy owls. The analyses were conducted separately for each of the five main prey groups: **a** bank voles, **b** *Microtus* voles, **c** shrews, **d** mice, and **e** birds. The relative vole abundance indices were used for bank voles and *Microtus* voles, whereas the pooled vole abundance index was used for shrews, mice, and birds. The interactions of autumn vole abundance with age and sex were kept in the models when presenting a significant *P* value or a trend (see Table 2). *N* = 629 food stores of 327 individuals of known sex and age

The number of the main prey species (bank voles and *Microtus* voles) stored by adult pygmy owls increased with vole abundance, but yearlings appeared to hoard a similar number of bank voles independent of vole abundance (Table 2 and Fig. 3). The results of the analyses for the total number of five main prey groups and their proportion were similar, showing consistent biological trends. The output of the latter is, therefore, presented in the Online Resource 3. Overall, females hoarded a higher number of small mammals (bank voles in high and low vole years, *Microtus* voles in high vole years, and shrews and mice in low vole years) than males (Table 2). Hoarding pygmy owls showed dietary shifts, because the number of birds, shrews, and mice in the food stores increased with lower autumn vole abundance in the environment, and was generally low in years of high vole abundance (Table 2 and Fig. 3). In low vole years, adult owls had a larger number of birds in the stores than yearlings, which instead had a higher number of bank voles, shrews, and mice (Table 2 and Fig. 3). Males showed a tendency to store a larger number of bird prey items than females (Table 2; as well as a higher proportion, see the analyses of the proportions of prey items, Online Resource 3).

**Table 2:** GLMMs analysing the variation in the number of prey items in pygmy owl food stores in relation to autumn vole abundance of the current year (vole index), and pygmy owl age and sex during 2003–2017

Prey group	Variable	Estimate ± SE		*χ*^2^	*P*
Bank voles	Intercept		1.253 ± 0.163		
	Age	Yearlings	1.091 ± 0.243	19.33	**< 0.0001**
		Adults	0 ± 0		
	Sex	Males	− 0.193 ± 0.116	2.79	0.0951
		Females	0 ± 0		
	Bank vole index		0.150 ± 0.027	29.50	**< 0.0001**
	Bank vole index × age	Yearlings	− 0.121 ± 0.040	9.04	**0.0026**
		Adults	0 ± 0		
	Removed term				
	Bank vole index × sex	Males	0.021 ± 0.038	0.320	0.5719
		Females	0 ± 0		
*Microtus* voles	Intercept		− 0.031 ± 0.186		
	Age	Yearlings	− 0.231 ± 0.159	2.11	0.1465
		Adults	0 ± 0		
	Sex	Males	− 0.588 ± 0.167	12.35	**0.0004**
		Females	0 ± 0		
	*Microtus vole* index		0.188 ± 0.012	248.65	**< 0.0001**
	Removed terms				
	*Microtus* vole index × age	Yearlings	0.019 ± 0.023	0.680	0.4094
		Adults	0 ± 0		
	*Microtus* vole index × sex	Males	0.002 ± 0.023	0.010	0.9244
		Females	0 ± 0		
Shrews	Intercept		0.795 ± 0.191		
	Age	Yearlings	0.874 ± 0.236	15.77	**0.0001**
		Adults	0 ± 0		
	Sex	Males	− 0.442 ± 0.163	7.32	**0.0068**
		Females	0 ± 0		
	Vole index		− 0.032 ± 0.013	5.58	**0.0182**
	Vole index × age	Yearlings	− 0.043 ± 0.018	5.82	**0.0158**
		Adults	0 ± 0		
	Removed term				
	Vole index × sex	Males	0.005 ± 0.017	0.09	0.7616
		Females	0 ± 0		
Mice	Intercept		− 0.089 ± 0.269		
	Age	Yearlings	1.292 ± 0.323	18.66	**< 0.0001**
		Adults	0 ± 0		
	Sex	Males	− 0.388 ± 0.211	3.38	0.0661
		Females	0 ± 0		
	Vole index		− 0.018 ± 0.018	0.76	0.3829
	Vole index × age	Yearlings	− 0.083 ± 0.024	11.71	**0.0006**
		Adults	0 ± 0		
	Removed term				
	Vole index × sex	Males	− 0.023 ± 0.024	0.94	0.3329
		Females	0 ± 0		
Birds	Intercept		0.782 ± 0.137		
	Age	Yearlings	− 0.277 ± 0.179	4.17	**0.0413**
		Adults	0 ± 0		
	Sex	Males	0.210 ± 0.122	2.97	0.0847
		Females	0 ± 0		
	Vole index		− 0.069 ± 0.010	38.15	**< 0.0001**
	Vole index × age	Yearlings	0.027 ± 0.014	3.53	0.0604
		Adults	0 ± 0		
	Removed term				
	Vole index × sex	Males	0.021 ± 0.013	2.430	0.1190
		Females	0 ± 0		

The analyses were conducted separately for the five main prey groups in the stores (bank voles, *Microtus* voles, shrews, mice, and small birds). The relative vole abundance indices were used for bank voles and *Microtus* voles, whereas the pooled vole abundance index was used for shrews, mice, and birds. Main terms were always kept in the models, while the interactions were kept only if significant (*P* < 0.05, in bold) or showing a closely significant trend (*P* < 0.06). Note that the estimates are at log scale. *N* = 629 food stores of 327 individuals of known sex and age. Individual identity of the owl and of the nest-box nested in the forest site were used as random effects

### Total response

**Fig. 4:**
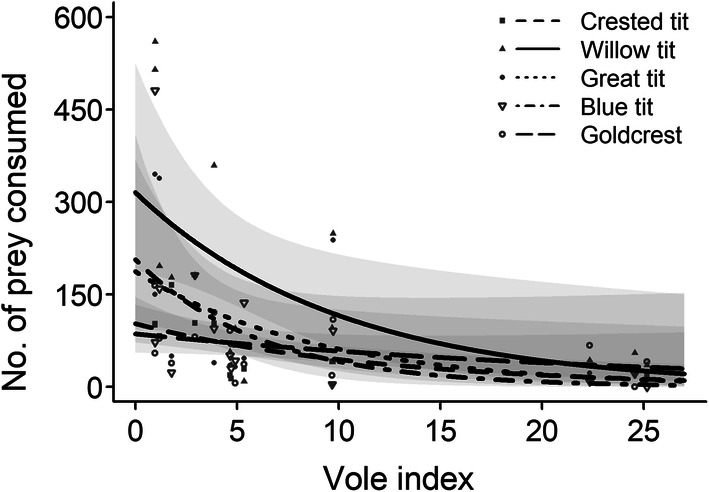
Predicted values (and 95% CI) of the number of consumed prey individuals by pygmy owls for the five species of birds (crested tit, willow tit, great tit, blue tit, and goldcrest) that were most frequently found in food stores during 2003–2017 in relation to the vole abundance in the autumn of the current year (‘Vole index’). All *P* values were significant (lower than 0.05), except for goldcrests that was 0.0895 (see Online Resource 2)

The estimated total prey biomass consumed by pygmy owls in a 2-month food-hoarding season averaged 68 kg during 2003–2017, and adult males consumed a larger proportion of it (approx. 30%, Table 3). During the course of the study, bank voles were the most frequently consumed prey item, followed by *Microtus* voles, and the consumption numbers of these voles were positively related to vole abundance (only a trend for bank voles, but significant for *Microtus* voles; Table 3). In years of high vole abundance, the amount of consumed birds was smaller than in low vole abundance years. Of the five most frequently stored bird species, the number of consumed crested, willow, great, and blue tits significantly increased with decreasing abundance of voles, whereas for the goldcrest, it was only a trend (Fig. 4 and Online Resource 2). The number of consumed willow tits was usually larger than that of other tit species and goldcrests (Online Resource 2).

**Table 3:** Estimated prey weight (kg) consumed by pygmy owls during the food-storing season, the percentage of biomass consumed by the age and sex classes, and the estimated number of the five most important prey groups consumed in the study area during 2003–17

Year	Prey weight	Percentage consumed by				Number consumed			
		Adults males	Adult females	Yearling males	Yearling females	*Microtus* voles	Shrews	Mice	Birds
2003	26.3	63.6	18.2	18.2	0.0	439	100	30	627
2004	81.0	14.8	17.3	26.6	41.3	427	1035	363	189
2005	64.8	25.9	37.1	22.2	14.8	965	886	676	1317
2006	30.9	38.9	53.4	7.8	0.0	0	178	0	2014
2007	122.3	19.6	17.6	35.3	27.4	300	799	549	334
2008	100.7	14.3	42.8	21.4	21.4	3118	492	170	106
2009	55.1	52.2	30.3	17.4	0.0	265	812	551	2185
2010	36.0	26.7	20.0	0.0	53.3	91	333	306	828
2011	134.3	14.3	5.4	37.5	42.9	3415	187	53	201
2012	33.6	64.3	21.4	14.3	0.0	125	923	249	1606
2013	72.0	10.0	6.7	26.7	56.7	324	1909	1100	352
2014	98.2	14.6	24.4	26.7	34.2	2972	96	80	152
2015	67.2	42.9	35.7	10.7	10.7	1194	400	101	319
2016	60.0	28.0	16.0	24.0	32.0	39	1550	190	903
2017	38.4	25.0	31.3	31.3	12.5	151	1037	191	453
Mean	68.1	30.3	25.2	21.3	23.2	922	716	307	772
S.D.	33.8	18.1	13.2	10.3	19.7	1211	537	301	694
slope						0.147 ± 0.019	− 0.035 ± 0.027	− 0.030 ± 0.035	− 0.110 ± 0.041
*χ*^2^						62.90	1.95	0.81	12.68
*P*						**< 0.0001**	0.1631	0.3691	**0.0004**

Estimates and standard errors (slope), and χ^2^ and *P* values for the GLMs for the numbers of prey animals consumed in relation with the vole abundance index are provided. The relative vole abundance indices were used for bank voles and *Microtus* voles, whereas the pooled bole abundance index was used for shrews, mice, and birds. Significant *P* values are highlighted in bold (*P* < 0.05)

## Discussion

We found age- and sex-related differences in both numerical responses and dietary shifts that reflect in the total response of the pygmy owls, suggesting that the impacts of this predator on the prey community might also vary with the fluctuating densities of their main prey (voles). The number of adult males hoarding food for the winter in the area remained relatively stable between years, while females and yearlings of both sexes showed pronounced numerical responses to fluctuations in vole abundance. Yearling owls hoarded mainly small mammals, whereas food store composition of adult owls varied more according to vole abundance, with a tendency to hoard more birds in years of low vole abundance. Females, furthermore, appeared to hoard more voles than males. Taken together, these sex- and age-specific differences in the numerical responses and dietary shifts produce differences in the total response of a predator, and, therefore, suggest that it could be important to consider the predator population structure instead of mere numbers of individuals when evaluating the impacts that a predator may have on prey community.

### Sex- and age-specific numerical responses

In agreement with our first prediction, numbers of adult males wintering in the study area varied less than those of adult females and yearlings of both sexes. This was expected, since adult males have not been usually observed during irruptive migratory movements (Lehikoinen et al. 2011a). In contrast, the numbers of adult females and yearlings were mainly determined by food availability in the beginning of the current hoarding season. The negative correlation in the residuals for the yearling models reflected the high among-year variability, likely explained by the cyclic fluctuations in vole populations. A relatively high abundance in the previous autumn and winter may lead to a high number of fledglings in the next spring, but thereafter be followed, in most years, by sudden declines of vole densities during the following summer (e.g., Korpimäki et al. 2005). During the winter months, in which competition for the limited resources is probably highest, more experienced and efficient hunters may be better able to persist and survive, while the others, facing a stronger starvation risk, may have to disperse from the area and/or perish.

Our interpretation of the numeric fluctuations of pygmy owls is supported by the fact that the majority of individuals captured during irruptive migrations at bird observatories are yearlings and females (Lehikoinen et al. 2011a), and the same pattern has also been found in other birds of prey. For example, in wintering European kestrels *Falco tinnunculus* (Village 1985) and in Tengmalm’s owls *Aegolius funereus* (Côté et al. 2007; Korpimäki and Hakkarainen 2012), the number of first-year individuals was lower and sex ratio was biased toward males when the main prey was less abundant. In great-horned owls (*Bubo virginianus*), floaters dispersed from the study area earlier than territorial older individuals during the decline phase of 10-year population cycle of the snow-shoe hares (*Lepus americanus*; Rohner 1996). Our results support the social-dominance hypothesis (Cox 1968) according to which dominant individuals (in our case adult males) are able to winter in the area. Remaining in the territory during the winter is of great importance for securing the breeding territory and nest cavity.

### Age and sex differences in dietary shifts

Our findings showed differences in the dietary shifts of age and sex classes of pygmy owls in relation to vole abundance in autumn. Consistent with our second prediction, yearling owls and females hoarded more small mammals (bank voles, *Microtus* voles, shrews, and mice) than adults and males, respectively. Adults of both sexes hoarded a larger amount of birds than yearlings when the main prey was scarce, and males had a tendency to hoard more birds than females. Differences between age classes were especially distinct in years of vole scarcity, but sex differences were present both in years of low and high vole abundance.

The age differences in the food store content of pygmy owls likely arise from lower hunting experience of yearlings compared to adults (Wunderle 1991), also supporting the hypothesis that they rely more on stored food (Masoero et al. 2018). In high vole abundance years, voles likely maximize energy intake of owls, since they are larger and heavier (Norrdahl and Korpimäki 2002), and require a less energy-consuming hunting technique than birds (i.e., long perching times close to the ground; Kullberg 1995). In low vole abundance years, the best option for inexperienced yearlings might be to store small mammals that are easier to capture than birds (Temeles 1985), even if less frequent to encounter in low years, whereas more experienced adults would be better at hunting birds (Sasvári et al. 2000; Rutz et al. 2006).

Females, being larger than males, are able to prey upon heavier species (*Microtus* voles). They likely rely more on stored food than males (Masoero et al. 2018) when they remain in the area, not only to survive the winter, but also to begin the breeding season in good body condition (Hirons 1985; Korpimäki 1987b). We suggest that male owls might be better able to rely on every-day hunting of avian prey that are available throughout the winter, consistent with the small male hypothesis put forward to explain the reversed sexual size dimorphism of birds of prey (Korpimäki and Hakkarainen 1991; Lee and Severinghaus 2004).

An question that rises from the results presented here and in Masoero et al. (2018) is: if adults are more experienced and better foragers than juveniles, why do they not hoard overall more food in the stores than yearlings? We suggest that the pygmy owls may be restricted in how much they can hunt and hoard without compromising their current health and survival. Adult males might invest less effort in food hoarding than females and yearlings, because they can afford doing so. Yearlings probably lack the plasticity in foraging behaviour shown by more experienced individuals, likely more able to adjust their foraging effort to the environmental conditions, as previously found also in European shags *Phalacrocorax aristotelis* (Daunt et al. 2007). Further examination of these questions would require data on the individual diet throughout the winter and on the calorific value of the different prey species. Nonetheless, the age- and sex-specific hoarding strategies observed here might be helpful for optimizing energy acquisition and expenditure, and for decreasing intraspecific food competition during winter (Newton 1979).

In this study, we used food store composition to characterize the diet composition of the pygmy owls. Although we cannot exclude that the diet of the pygmy owls might present slight differences from the food store composition, the large extent of the data set in terms of years and of numbers of food stores examined allows us to suggest that these results likely reflect the differences in among-year dietary shifts between age and sex classes. Previous studies showed that functional responses could vary between sexes (hemipteran, *Lyctocoris campestris*; Parajulee et al. 1994) and according to social status (solitary individuals and social groups in Eurasian lynx *Lynx lynx*; Nilsen et al. 2009). Our study, however, combines the analysis of the age and sex differences in the functional response to analysis of the numerical response.

### Total response of pygmy owls

In agreement with our third prediction, the total response of pygmy owls and the amount of consumed prey can change during their lifetime (age effect), according to the sex of the individual and in relation to the abundance of the main prey. The different age and sex classes of a single predator species appear thus to act functionally as different predator types (‘trophic species’, as defined by Sih et al. 1998). In addition, the diet of a predator can also be altered by the density of the main prey, as shown here and in previous studies (see, e.g., Korpimäki and Norrdahl 1989, 1991; Nielsen 1999; Therrien et al. 2014; Liu et al. 2018). Age and sex segregation in foraging could mean a lower impact on the population of the single species of prey, especially in the context of fluctuating abundance of the main prey. When the main prey is abundant, predator numbers can increase and most alternative prey species would be released from predation. On the other hand, when the main prey is scarce, the species-specific consumption would be higher, but the prey community as a whole would benefit by an overall decrease in predation pressure, due both to the lower number of wintering individuals and to the predation pressure being shared among a larger amount of prey species.

In our study area, the food stores of pygmy owls mainly consisted of small mammals, in particular bank voles, similar to other north-European studies (Mikkola 1983; Solheim 1984a; Halonen et al. 2007; see Online Resource 1). *Microtus* voles were present with high numbers in the food stores only at their peak density years, whereas bank voles were stored both during high and low vole abundance years. When vole abundance was low, shrews, mice and birds were the main alternative prey of pygmy owls. In these years, predation on some of the bird species appeared substantial, since wintering owls, in particular adult males that still remained resident in the study area, shifted to small birds. The most abundant bird species in the stores (willow, crested, great, and blue tits and the goldcrest) are also the ones most often observed during mist-net censuses in winter in the forests of the study area (Morosinotto et al. 2017b). Both Ekman (1986) and Kullberg (1995) have reported larger proportions of birds in the diet compared to what we observed in the food stores. Indeed, it may not be necessary to store large quantities of birds, since their capture later during the winter is not affected by the snow cover that protects small mammals from pygmy owls and other avian predators (Sonerud 1986; Halonen et al. 2007). In these conditions, however, it is likely that the stored food is especially important for the survival of pygmy owls, considering also the high overlap of winter diets with a larger competitor, the Tengmalm’s owl (Suhonen et al. 2007; Korpimäki and Hakkarainen 2012).

Quantifying the total response of pygmy owls is a challenging task because of a lack of data on the real numbers of prey species available in the field on one hand and incomplete information on the amount of food stored in undiscovered stores in natural tree cavities on the other. In our study area, natural cavities in trees were only very seldom used as food stores (Baroni et al. 2020). The obtained result is thus an approximation of the real consumption by pygmy owls on these species, but we consider that it anyway reflects inter-specific differences between prey species and its among-year variation in relation to the vole abundance. Further studies should investigate in more detail how this variation in consumption rate could impact prey populations and how much alternative prey species could be detrimentally impacted by the sex- and age-specific limiting actions of a predator depending on the main prey.

## Conclusions

Our study addresses how traits of a predator, such as age and sex, are shaping its numerical, functional, and total responses to fluctuating abundance of main prey in winter. The age and sex composition of the pygmy owl population showed large spatio-temporal variation in boreal forests. Rapid numerical response of pygmy owls to fluctuations of main prey abundance (voles) is brought by increasing numbers of yearling owls and adult females that mainly consume voles, whereas functional response is induced by resident adult males that are able to shift to small birds in years of vole scarcity. These dietary shifts of adult males induce increasing consumption of small birds in years of vole scarcity.

## Electronic supplementary material

Below is the link to the electronic supplementary material.

Supplementary material 1 (DOCX 43 kb)

## References

[CR1] Abrams PA (2000). The evolution of predator-prey interactions: theory and evidence. Annu Rev Ecol Syst.

[CR2] Barbaro L, Blache S, Trochard G (2016). Hierarchical habitat selection by Eurasian pygmy owls *Glaucidium passerinum* in old-growth forests of the southern French Prealps. J Ornithol.

[CR3] Baroni D, Korpimäki E, Selonen V, Laaksonen T (2020). Tree cavity abundance and beyond: nesting and food storing sites of the pygmy owl in managed boreal forests. For Ecol Manag.

[CR4] Bates D, Maechler M, Bolker B, Walker S (2015). Fitting Linear Mixed-Effects Models using lme4. R package v. 1.1-15. J Stat Softw.

[CR5] Côté M, Ibarzabal J, St-Laurent M-H (2007). Age-dependent response of migrant and resident *Aegolius* owl species to small rodent population fluctuations in the Eastern Canadian boreal forest. J Raptor Res.

[CR6] Cox GW (1968). The role of competition in the evolution of migration. Evolution (N Y).

[CR7] Curio E (1983). Why do young birds reproduce less well?. Ibis.

[CR8] Daunt F, Wanless S, Harris MP (2007). Older and wiser: improvements in breeding success are linked to better foraging performance in European shags. Funct Ecol.

[CR9] Earhart CM, Johnson NK (1970). Size dimorphism and food habits of North American owls. Condor.

[CR10] Ekman J (1986). Tree use and predator vulnerability of wintering passerines. Ornis Scand.

[CR11] Forslund P, Pärt T (1995). Age and reproduction in birds - hypotheses and tests. Trends Ecol Evol.

[CR12] Glutz von Blotzheim UN, Bauer KM (1980). Handbuch der Vögel Mitteleuropas.

[CR13] Hakkarainen H, Korpimäki E (1991). Reversed sexual size dimorphism in Tengmalm’s owl: is small male size adaptive?. Oikos.

[CR14] Halonen M, Mappes T, Meri T, Suhonen J (2007). Influence of snow cover on food hoarding in pygmy owls *Glaucidium passerinum*. Ornis Fenn.

[CR15] Hansson L, Henttonen H (1988). Rodent dynamics as community process. Trends Ecol Evol.

[CR16] Hirons GJM (1985). The importance of body reserves for successful reproduction in the Tawny owl (*Strix aluco*). J Zool.

[CR17] Huitu O, Norrdahl K, Korpimäki E (2003). Landscape effects on temporal and spatial properties of vole population fluctuations. Oecologia.

[CR18] Kellomäki E (1977). Food of the pygmy owl *Glaucidium passerinum* in the breeding season. Ornis Fenn.

[CR19] Keynan O, Yosef R (2010). Temporal changes and sexual differences of impaling behavior in Southern grey shrike (*Lanius meridionalis*). Behav Processes.

[CR20] Korpimäki E (1987). Dietary shifts, niche relationships and reproductive output of coexisting kestrels and long-eared owls. Oecologia.

[CR21] Korpimäki E (1987). Breeding performance of Tengmalm’s owl *Aegolius funereus*: effects of supplementary feeding in a peak vole year. Ibis.

[CR22] Korpimäki E (1992). Fluctuating food abundance determines the lifetime reproductive success of male Tengmalm’s owls. J Anim Ecol.

[CR23] Korpimäki E, Hakkarainen H (1991). Fluctuating food supply affects the clutch size of Tengmalm’s owl independent of laying date. Oecologia.

[CR24] Korpimäki E, Hakkarainen H (2012). The boreal owl: ecology, behaviour, and conservation of a forest-dwelling predator.

[CR25] Korpimäki E, Norrdahl K (1989). Predation of Tengmalm’s owls: numerical responses, functional responses and dampening impact on population fluctuations of microtines. Oikos.

[CR26] Korpimäki E, Norrdahl K (1991). Numerical and functional responses of kestrels, short-eared owls, and long-eared owls to vole densities. Ecology.

[CR27] Korpimäki E, Norrdahl K, Rinta-Jaskari T (1991). Responses of stoats and least weasels to fluctuating food abundances: is the low phase of the vole cycle due to mustelid predation?. Oecologia.

[CR28] Korpimäki E, Norrdahl K, Huitu O, Klemola T (2005). Predator-induced synchrony in population oscillations of coexisting small mammal species. Proc R Soc B Biol Sci.

[CR29] Krebs CJ, Myers JH (1978). Population cycles in small mammals. Adv Ecol Res.

[CR30] Kullberg C (1995). Strategy of the pygmy owl while hunting avian and mammalian prey. Ornis Fenn.

[CR31] Lagerström M, Syrjänen J (1990). Varpuspöllön iän määrittäminen (Summary: ageing pygmy owls). Lintumies.

[CR32] Lee Y-F, Severinghaus LL (2004). Sexual and seasonal differences in the diet on Lanyu scops owls based on fecal analysis. J Wildl Manag.

[CR33] Lehikoinen A, Hokkanen T, Lokki H (2011). Young and female-biased irruptions in pygmy owls *Glaucidium passerinum* in southern Finland. J Avian Biol.

[CR34] Lehikoinen A, Ranta E, Pietiäinen H (2011). The impact of climate and cyclic food abundance on the timing of breeding and brood size in four boreal owl species. Oecologia.

[CR35] Liu D, Guo X, Zhong D (2018). Prey density and a conspecific competitor influence multiple predator effects in a crab clam foraging system. Aquaculture.

[CR36] Marchetti K, Price T (1989). Differences in the foraging of juvenile and adult birds: the importance of developmental constraints. Biol Rev.

[CR37] Masoero G, Morosinotto C, Laaksonen T, Korpimäki E (2018). Food hoarding of an avian predator: sex- and age-related differences under fluctuating food conditions. Behav Ecol Sociobiol.

[CR38] Mikkola H (1983). Owls of Europe.

[CR39] Morosinotto C, Villers A, Thomson RL (2017). Competitors and predators alter settlement patterns and reproductive success of an intraguild prey. Ecol Monogr.

[CR40] Morosinotto C, Villers A, Varjonen R, Korpimäki E (2017). Food supplementation and predation risk in harsh climate: interactive effects on abundance and body condition of tit species. Oikos.

[CR41] Newton I (1979). Population Ecology of Raptors.

[CR42] Newton I, Marquiss M (1982). Food, predation and breeding season in Sparrowhawks (*Accipiter nisus*). J Zool.

[CR43] Nielsen OK (1999). Gyrfalcon predation on ptarmigan: numerical and functional responses. J Anim Ecol.

[CR44] Nilsen EB, Linnell JDC, Odden J, Andersen R (2009). Climate, season, and social status modulate the functional response of an efficient stalking predator: the Eurasian lynx. J Anim Ecol.

[CR45] Norrdahl K, Korpimäki E (2002). Changes in individual quality during a 3-year population cycle of voles. Oecologia.

[CR46] O’Donoghue M, Boutin S, Krebs CJ, Hofer EJ (1997). Numerical responses of coyotes and lynx to the snowshoe hare cycle. Oikos.

[CR47] O’Donoghue M, Boutin S, Krebs CJ (1998). Functional responses of coyotes and lynx to the snowshoe hare cycle. Ecology.

[CR48] Oaten A, Murdoch WW (1975). Functional response and stability in predator-prey systems. Am Nat.

[CR49] Parajulee MN, Phillips TW, Hogg DB (1994). Functional response of Lyctocoris campestris (F.) adults: effects of predator sex, prey species, and experimental habitat. Biol Control.

[CR67] R Core Team (2019) R: A language and environment for statistical computing. R Foundation for Statistical Computing

[CR50] Rohner C (1996). The numerical response of great horned owls to the snowshoe hare cycle: consequences of non-territorial “floaters” on demography. J Anim Ecol.

[CR51] Rutz C, Whittingham MJ, Newton I (2006). Age-dependent diet choice in an avian top predator. Proc R Soc B Biol Sci.

[CR52] Salamolard M, Butet A, Leroux A, Bretagnolle V (2000). Response of an avian predator to variation in prey density at a temperate latitude. Ecology.

[CR53] Salo P, Banks PB, Dickman CR, Korpimäki E (2010). Predator manipulation experiments: impacts on populations of terrestrial vertebrate prey. Ecol Monogr.

[CR54] Sasvári L, Hegyi Z, Csörgõ T, Hahn I (2000). Age-dependent diet change, parental care and reproductive cost in tawny owls *Strix aluco*. Acta Oecologica.

[CR55] Scherzinger W (1970) Zum Aktionssystem des Sperlingskauzes (*Glaucidium passerinum*, L.). Zoologica 118:1–120

[CR56] Schönn S (1980) Der Sperlingskauz. Die Neue Brehm-Bücherei. A. Ziemsen Verlag, Wittenberg Lutherstadt

[CR57] Schulenburg J, Wiesner J (1986). Zur Winternahrung des Sperlinkgskauzes (*Glaucidium passerinum*) in zwei unterschiedlichen Gebieten der DDR. Acta Ornithoecol.

[CR58] Sih A, Englund G, Wooster D (1998). Emergent impacts of multiple predators on prey. Trends Ecol Evol.

[CR59] Skaug H, Fournier D, Bolker B, et al (2016) generalized linear mixed models using “AD Model Builder”. R package v. 0.8.3.3

[CR60] Solheim R (1984). Caching behaviour, prey choice and surplus killing by pygmy owls *Glaucidium passerinum* during winter, a functional response of a generalist predator. Ann Zool Fennici.

[CR61] Solheim R (1984). Breeding biology of the pygmy owl Glaucidium passerinum in two biogeographical zones in southeastern Norway. Ann Zool Fenn.

[CR62] Solomon ME (1949). The natural control of animal populations. J Anim Ecol.

[CR63] Sonerud GA (1986). Effect of snow cover on seasonal changes in diet, habitat, and regional distribution of raptors that prey on small mammals in boreal zones of Fennoscandia. Ecography.

[CR64] Strom H, Sonerud GA (2001). Home range and habitat selection in the pygmy owl *Glaucidium passerinum*. Ornis Fenn.

[CR65] Suhonen J, Halonen M, Mappes T (1993). Predation risk and the organisation of the *Parus guild*. Oikos.

[CR66] Suhonen J, Halonen M, Mappes T, Korpimäki E (2007). Interspecific competition limits larders of pygmy owls *Glaucidium passerinum*. J Avian Biol.

[CR68] Temeles EJ (1985). Sexual size dimorphism of bird-eating hawks: the effect of prey vulnerability. Am Nat.

[CR69] Terraube J, Villers A, Poudré L (2017). Increased autumn rainfall disrupts predator-prey interactions in fragmented boreal forests. Glob Chang Biol.

[CR70] Therrien J-F, Gauthier G, Korpimäki E, Bêty J (2014). Predation pressure by avian predators suggests summer limitation of small-mammal populations in the Canadian Arctic. Ecology.

[CR71] Vermeij GJ (1994). The evolutionary interaction among species: selection, escalation, and coevolution. Annu Rev Ecol Syst.

[CR72] Village A (1985). Turnover, age and sex-ratios of kestrels (*Falco tinnunculus*) in South Scotland. J Zool.

[CR73] Winnie J, Creel S (2017). The many effects of carnivores on their prey and their implications for trophic cascades, and ecosystem structure and function. Food Webs.

[CR74] Wunderle JM (1991). Age-specific foraging proficiency in birds. Curr Ornithol.

[CR75] Zuur AF, Ieno EN, Walker NJ (2009). Mixed effects models and extensions in ecology with R.

