# Supplementary material for: Age and sex differences in numerical responses, dietary shifts, and total responses of a generalist predator to population dynamics of main prey
Source: Oecologia. 2020 Feb 1;192(3):699–711. doi: 10.1007/s00442-020-04607-x (PMC7058601; doi:10.1007/s00442-020-04607-x)
Supplement: Supplementary file 1 — Supplementary material 1 (DOCX 43 kb) [file 442_2020_4607_MOESM1_ESM.docx]

**Electronic Supplementary Materials**

**Age and sex differences in numerical responses, dietary shifts and total responses of a generalist predator to population dynamics of main prey**

Giulia Masoero^1^, Toni Laaksonen^1,2^, Chiara Morosinotto^1,3^, Erkki Korpimäki^1^

^1^ Section of Ecology, Department of Biology, FI-20014 University of Turku, Finland

^2^ Natural Resources Institute Finland (Luke), Turku, Finland

^3^ Novia University of Applied Sciences, Bioeconomy research team, Raseborgsvägen 9, FI-10600 Ekenäs, Finland

Correspondence: Giulia Masoero giulia.masoero@gmail.com

**Online Resource 1**

The total number (No.), proportion (%) and average body weight (± SD; g) of different prey species (number of individuals that were whole and measured = n) in food stores of pygmy owls during 2003-17. The body weight of the prey species was reported with the measured value (if n = 1), average value (mean ± SD; if n > 1), or empty (-) when prey items where not weighted.

| **English name** | **Scientific name** | **No.** | | **%** | | **Weight (mean** ± **SD (n))** | |  |
| --- | --- | --- | --- | --- | --- | --- | --- | --- |
| Eurasian pygmy shrew | *Sorex minutus* | 465 | | 2.61 | | 3.1 ± 0.7 (385) | |  |
| Eurasian shrew | *S. araneus* | 1437 | | 8.06 | | 5.7 ± 1.1 (1118) | |  |
| Even-toothed Shrew | *S. isodon* | 5 | | 0.03 | | 4.9 ± 1.7 (5) | |  |
| Eurasian water shrew | *Neomys fodiens* | 7 | | 0.04 | | 12.7 ± 1 (6) | |  |
| Bank vole | *Myodes glareolus* | 8560 | | 47.99 | | 15.4 ± 3.2 (7106) | |  |
| Field vole | *Microtus agrestis* | 2038 | | 11.43 | | 18.9 ± 5.3 (1808) | |  |
| Sibling vole | *M. rossiaemeridionalis* | 3166 | | 17.75 | | 20.1 ± 4.7 (2844) | |  |
| Wood lemming | *Myopus schisticolor* | 2 | | 0.01 | | - | |  |
| Brown rat | *Rattus norvegicus* | 1 | | 0.01 | | - | |  |
| Eurasian harvest mouse | *Micromys minutus* | 815 | | 4.57 | | 6.1 ± 1.7 (645) | |  |
| House mouse | *Mus musculus* | 53 | | 0.30 | | 13.9 ± 6.3 (47) | |  |
| Unidentified *microtus* | *Microtus sp* | 28 | | 0.16 | | - | |  |
| Unidentified vole | *M. glareolus/Microtus sp* | 6 | | 0.03 | | 8.5 ± 0.7 (2) | |  |
| Mammals total | | | 16583 | | 92.96 | |  | |
|  |  |  | |  | |  | |  |
| Lesser spotted woodpecker | *Dryobates minor* | 3 | | 0.02 | | 20.8 ± 0.4 (2) | |  |
| Meadow pipit | *Anthus pratensis* | 1 | | 0.01 | | - | |  |
| White wagtail | *Motacilla alba* | 1 | | 0.01 | | - | |  |
| Bohemian waxwing | *Bombycilla garrulus* | 2 | | 0.01 | | - | |  |
| Dunnock | *Prunella modularis* | 1 | | 0.01 | | 18.5 | |  |
| European robin | *Erithacus rubecula* | 6 | | 0.03 | | 16.9 ± 1.8 (5) | |  |
| Fieldfare | *Turdus pilaris* | 1 | | 0.01 | | - | |  |
| Song thrush | *T. philomelos* | 3 | | 0.02 | | - | |  |
| Redwing | *T. iliacus* | 30 | | 0.17 | | - | |  |
| Lesser whitethroat | *Sylvia curruca* | 1 | | 0.01 | | 11.5 | |  |
| Goldcrest | *Regulus regulus* | 163 | | 0.91 | | 5.4 ± 0.5 (142) | |  |
| Long-tailed Tit | *Aegithalos caudatus* | 16 | | 0.09 | | 9 ± 0.8 (15) | |  |
| Willow tit | *Poecile montanus* | 299 | | 1.68 | | 11.2 ± 1 (246) | |  |
| Crested tit | *Lophophanes cristatus* | 130 | | 0.73 | | 11.4 ± 0.9 (100) | |  |
| Coal tit | *Periparus ater* | 19 | | 0.11 | | 8.9 ± 0.7 (18) | |  |
| Eurasian blue tit | *Cyanistes caeruleus* | 148 | | 0.83 | | 11.4 ± 1.4 (133) | |  |
| Great tit | *Parus major* | 197 | | 1.10 | | 19.1 ± 1.7 (159) | |  |
| Eurasian treecreeper | *Certhia familiaris* | 44 | | 0.25 | | 8.9 ± 0.7 (36) | |  |
| House sparrow | *Passer domesticus* | 12 | | 0.07 | | 34.6 ± 1.4 (9) | |  |
| Eurasian tree sparrow | *P. montanus* | 5 | | 0.03 | | 23.5 ± 1.5 (4) | |  |
| Common chaffinch | *Fringilla coelebs* | 6 | | 0.03 | | 20.9 ± 4.1 (4) | |  |
| Brambling | *F. montifringilla* | 1 | | 0.01 | | - | |  |
| European greenfinch | *Carduelis chloris* | 38 | | 0.21 | | 28.3 ± 2.5 (33) | |  |
| Eurasian siskin | *C. spinus* | 4 | | 0.02 | | 13.3 ± 3.9 (4) | |  |
| Redpoll | *C. flammea* | 61 | | 0.34 | | 14.1 ± 2.9 (49) | |  |
| Two-barred crossbill | *Loxia leucoptera* | 1 | | 0.01 | | 23 | |  |
| Red crossbill | *L. curvirostra* | 11 | | 0.06 | | 40.5 ± 0.7 (2) | |  |
| Parrot crossbill | *L. pytyopsittacus* | 3 | | 0.02 | | - | |  |
| Red/parrot crossbill | *L. curvirostra/pytyopsittacus* | 3 | | 0.02 | | - | |  |
| Eurasian bullfinch | *Pyrrhula pyrrhula* | 20 | | 0.11 | | 30.7 ± 3.5 (11) | |  |
| Yellowhammer | *Emberiza citrinella* | 22 | | 0.12 | | 30.8 ± 2.3 (19) | |  |
|  | *Passeriformes sp.* | 3 | | 0.02 | | - | |  |
| Birds total | | | 1255 | | 7.04 | |  | |
|  |  |  | |  | |  | |  |
| **Total** |  | **17838** | | **100.00** | |  | |  |

Previous studies on pygmy owl caches in North Europe also reported high numbers of bank voles and *Microtus* voles, and low numbers of shrews, mice and birds (Mikkola 1983; Solheim 1984; Halonen et al. 2007). *Microtus* voles were present with high numbers in the food stores only at their peak density years, whereas bank voles were stored both during high and low vole abundance years. Even if in low numbers, bank voles are more conspicuous, moving more above the snow, usually in forests, and often climb on trees in winter, whereas *Microtus* voles mainly occupy sub-nivean space of open areas in winter (Pulliainen and Keränen 1979; Hansson 1982). The proportion of birds observed was very low in comparison to previous literature on pygmy owl diet, but it has to be considered that, among all previous studies, those reporting higher proportions of birds were either done during the breeding season (Kellomäki 1977; Mikkola 1983) or in Central and South Europe (Schulenburg and Wiesner 1986; Bonvicini and Della Ferrera 1994; Boiko and Shutova 2005; Muller and Riols 2013; Šotnár et al. 2015). In Northern Europe, the density of small passerines is low in winter due to post-breeding migration, and comparisons are difficult. Among the studies on wintering pygmy owls in Northern Europe, Solheim (1984b; South-eastern Norway) and Mikusek et al. (2001; White Sea, Russia) reported similar percentages in food stores (respectively, 11% and 9% of birds), whereas in Finland Mikkola (1983) and Järvi (1986) report percentages closer to the ones in spring (32% and 44%, respectively). Ekman (1986; South-western Sweden) analysed content of regurgitated pellets in winter and recorded around 51% of birds.

**References**

Boiko NS, Shutova E V (2005) Diets of the pygmy owl *Glaucidium Passerinum* and Tengmalm’s owl *Aegolius Funereus* in the Gulf of Kandalaksha area, White Sea. Status Raptor Popul East Fennoscandia 23–29

Bonvicini P, Della Ferrera E (1994) Dati sull’alimentazione della Civetta nana *Glaucidium passerinum* nelle Alpi Orobie (SO). In: Atti del 6° Convegno Italiano di Ornitologia (Torino, 8-11 ottobre 1991). Museo regionale di Scienze Naturali di Torino, Torino, pp 431–432

Halonen M, Mappes T, Meri T, Suhonen J (2007) Influence of snow cover on food hoarding in pygmy owls *Glaucidium passerinum*. Ornis Fenn 84:105–111

Hansson L (1982) Use of forest edges by Swedish Mammals. Fauna och Flora 77:301–308

Järvi E (1986) Varpuspöllön Glaucidium passerinum talvivarastojen karttuminen ja käyttö talvikaudella 1985/86. Ornis Botnica 8:4–26

Kellomäki E (1977) Food of the pygmy owl *Glaucidium passerinum* in the breeding season. Ornis Fenn 54:1–29

Mikkola H (1983) Owls of Europe. T. and A. D. Poyser

Mikusek R, Kloubec B, Obuch J (2001) Diet of the pygmy owl (*Glaucidium passerinum*) in eastern Central Europe. Buteo 12:47–60

Muller Y, Riols C (2013) Premières données sur le régime alimentaire de la Chevêchette dʼEurope *Glaucidium passerinum* dans les Vosges du Nord. Ciconia 37:107–113

Muller Y, Riols C (2013) Premières données sur le régime alimentaire de la Chevêchette dʼEurope *Glaucidium passerinum* dans les Vosges du Nord. Ciconia 37:107–113

Schulenburg J, Wiesner J (1986) Zur Winternahrung des Sperlinkgskauzes (*Glaucidium passerinum*) in zwei unterschiedlichen Gebieten der DDR. Acta Ornithoecol 1:167–183

Solheim R (1984) Caching behaviour, prey choice and surplus killing by pygmy owls *Glaucidium passerinum* during winter, a functional response of a generalist predator. Ann Zool Fennici 21:301–308

Šotnár K, Pačenovský S, Obuch J (2015) On the food of the Eurasian pygmy owl (*Glaucidium passerinum*) in Slovakia. Slovak Raptor J 9:115–126. doi: 10.1515/srj-2015-0009.

**Online Resource 2**

We estimated the total number of prey items consumed by pygmy owls for the five species of birds most commonly found in the food stores in winter. The species were: great tit (*Parus major*), blue tit (*Cyanistes caeruleus*), crested tit (*Lophophanes cristatus*), willow tit (*Poecile montanus*) and goldcrest (*Regulus regulus*).

The estimate was calculated with the formula of Korpimäki and Norrdahl (1989). First we calculated the number of prey animals (NPA) consumed by pygmy owls during the food storing season:

$$NPA= \frac{C_{AM}\times{PPA}_{AM}+C_{AF}\times{PPA}_{AF}+C_{YM}\times{PPA}_{YM}+C_{YF}\times{PPA}_{YF}}{MWPA}$$

where

$C_{age-sex}$ = consumption (g) of the age and sex class during the food hoarding season = (number of individuals of that age and sex class $\times$ daily food requirement of that sex class $\times$ length of the food hoarding season (60 days)).

Daily food requirement was estimated as 40 g per day for males and 45 g per day for females. Data on daily food requirements was obtained from Glutz von Blotzheim and Bauer (1980), where the mean daily consumption outside the breeding season was 30 g and increased to 40 g in females before egg-laying. During the low temperatures (-10° C), the daily food consumption was twice as large as during positive temperatures (Scherzinger 1970).

${PPA}_{age-sex}$ = percentage of prey group biomass in the food stores of that age and sex class during the food hoarding season.

$MWPA$ = mean weight (g) of prey animals (see Online Resource 1).

Estimated number of crested tits, willow tits, great tits, blue tits and goldcrests consumed by pygmy owls during the food storing seasons of 2003-17. Estimate and standard error (slope), χ² and p-value for the GLMs for the numbers of prey animals consumed in relation with the vole abundance index are provided.

| **year** | **Crested tit** | **Willow tit** | **Great tit** | **Blue tit** | **Goldcrest** |
| --- | --- | --- | --- | --- | --- |
| 2003 | 165 | 178 | 50 | 23 | 39 |
| 2004 | 41 | 96 | 3 | 4 | 19 |
| 2005 | 41 | 249 | 238 | 91 | 109 |
| 2006 | 100 | 560 | 345 | 72 | 55 |
| 2007 | 68 | 95 | 32 | 43 | 6 |
| 2008 | 18 | 36 | 11 | 0 | 41 |
| 2009 | 83 | 196 | 338 | 159 | 79 |
| 2010 | 104 | 359 | 39 | 94 | 121 |
| 2011 | 30 | 55 | 22 | 21 | 0 |
| 2012 | 103 | 514 | 150 | 481 | 164 |
| 2013 | 14 | 36 | 47 | 30 | 70 |
| 2014 | 11 | 43 | 23 | 9 | 68 |
| 2015 | 19 | 57 | 35 | 51 | 92 |
| 2016 | 104 | 181 | 132 | 181 | 81 |
| 2017 | 29 | 9 | 46 | 136 | 37 |
| Mean | 61 | 178 | 101 | 93 | 65 |
| S.D. | 45 | 175 | 116 | 122 | 45 |
| slope | -0.084 ± 0.027 | -0.100 ± 0.043 | -0.111 ± 0.061 | -0.161 ± 0.077 | -0.039 ± 0.025 |
| χ² | 14.41 | 8.95 | 8.72 | 9.50 | 2.88 |
| p | **0.0001** | **0.0028** | **0.0159** | **0.0021** | 0.0895 |

**References**

Glutz von Blotzheim UN, Bauer KM (1980) Handbuch der Vögel Mitteleuropas. AULA-Verlag Wiesbaden

Korpimäki E, Norrdahl K (1989) Predation of Tengmalm’s owls: Numerical responses, functional responses and dampening impact on population fluctuations of microtines. Oikos 54:154. doi: 10.2307/3565261

Scherzinger W (1970) Zum Aktionssystem des Sperlingskauzes (*Glaucidium passerinum*, L.). E. Schweizerbart

**Online Resource 4**

GLMMs analysing the variation in the proportion of prey items pygmy owl food stores in relation to autumn vole abundance of the current year (vole index), pygmy owl age (Y = yearlings, A = adults) and sex (M = males, F = females) during 2003-2017. The analyses were conducted separately for the five main prey groups in the stores (bank voles, *Microtus* voles, shrews, mice and small birds). Main terms were always kept in the models to control for the effect, while the interactions were kept only if significant (P<0.05, in bold) or showing an almost significant trend (P<0.06). Note that the estimates are at log-scale. N = 629 food stores of 327 individuals. Individual identity of the owl and of the box nested in the forest-site were used as random effects.

| **Prey group** | **Variable** | | **Estimate ± SE** | | | **χ²** | | **P** | |
| --- | --- | --- | --- | --- | --- | --- | --- | --- | --- |
| **Bank voles** | | Intercept | |  | -0.136 ± 0.193 |  |  | |  |
|  | | Age | | Y  A | 1.492 ± 0.257  0 ± 0 | 39.74 | <**0.0001** | |  |
|  | | Sex | | M  F | -0.336 ± 0.236  0 ± 0 | 0.40 | 0.5292 | |  |
|  | | Vole index | |  | -0.052 ± 0.028 | 12.81 | **0.0003** | |  |
|  | | Vole index X Age | | Y  A | -0.143 ± 0.041  0 ± 0 | 12.31 | **0.0005** | |  |
|  | | Vole index X Sex | | M  F | 0.078 ± 0.033  0 ± 0 | 5.56 | **0.0183** | |  |
| ***Microtus* voles** | | Intercept | |  | -3.279 ± 0.178 |  |  | |  |
|  | | Age | | Y  A | -0.690 ± 0.134  0 ± 0 | 26.35 | <**0.0001** | |  |
|  | | Sex | | M  F | -0.373 ± 0.185  0 ± 0 | 4.05 | **0.0441** | |  |
|  | | Vole index | |  | 0.231 ± 0.010 | 497.69 | <**0.0001** | |  |
|  | | *Removed term* | |  | |  |  | |  |
|  | | Vole index X Age | | Y  A | 0.030 ± 0.020  0 ± 0 | 2.14 | 0.1436 | |  |
|  | | Vole index X Sex | | M  F | -0.013 ± 0.020  0 ± 0 | 0.47 | 0.4923 | |  |
| **Shrews** | | Intercept | |  | -1.631 ± 0.161 |  |  | |  |
|  | | Age | | Y  A | 0.035 ± 0.135  0 ± 0 | 0.07 | 0.7928 | |  |
|  | | Sex | | M  F | -0.231 ± 0.166  0 ± 0 | 1.95 | 0.1626 | |  |
|  | | Vole index | |  | -0.080 ± 0.008 | 114.67 | <**0.0001** | |  |
|  | | *Removed term* | |  |  |  |  |  |  |
|  | | Vole index X Age | | Y  A | -0.023 ± 0.016  0 ± 0 | 2.03 | 0.1538 | |  |
|  | | Vole index X Sex | | M  F | 0.006 ± 0.015  0 ± 0 | 0.18 | 0.6710 | |  |
| **Mice** | | Intercept | |  | -3.483 ± 0.257 |  |  | |  |
|  | | Age | | Y  A | 0.940 ± 0.286  0 ± 0 | 1.77 | 0.1840 | |  |
|  | | Sex | | M  F | -0.256 ± 0.228  0 ± 0 | 1.27 | 0.2606 | |  |
|  | | Vole index | |  | -0.049 ± 0.015 | 62.79 | <**0.0001** | |  |
|  | | Vole index X Age | | Y  A | -0.070 ± 0.022  0 ± 0 | 10.09 | **0.0015** | |  |
|  | | *Removed term* | |  | |  |  | |  |
|  | | Vole index X Sex | | M  F | -0.027 ± 0.021  0 ± 0 | 1.63 | 0.2010 | |  |
| **Birds** | | Intercept | |  | -1.445 ± 0.199 |  |  | |  |
|  | | Age | | Y  A | -1.180 ± 0.245  0 ± 0 | 7.34 | **0.0067** | |  |
|  | | Sex | | M  F | 0.627 ± 0.198  0 ± 0 | 10.08 | **0.0015** | |  |
|  | | Vole index | |  | -0.152 ± 0.014 | 145.69 | <**0.0001** | |  |
|  | | Vole index X Age | | Y  A | 0.078 ± 0.019  0 ± 0 | 16.35 | **0.0001** | |  |
|  | | *Removed term* | |  | |  |  | |  |
|  | | Vole index X Sex | | M  F | 0.031 ± 0.017  0 ± 0 | 3.19 | 0.0740 | |  |
